# Tumor-Infiltrating CD8+ T Cells Driven by the Immune Checkpoint-Associated Gene IDO1 Are Associated With Cervical Cancer Prognosis

**DOI:** 10.3389/fonc.2021.720447

**Published:** 2021-10-27

**Authors:** Shun Zhang, Junhui Wan, Minjie Chen, Desheng Cai, Junlan Xu, Qi Chen

**Affiliations:** ^1^ General Surgery Department, The First Affiliated Hospital of Nanchang University, Nanchang, China; ^2^ Obstetrics and Gynaecology Department, The First Affiliated Hospital of Nanchang University, Nanchang, China; ^3^ Queen Mary College, Nanchang University, Nanchang, China; ^4^ Obstetrics and Gynecology Department, The First Affiliated Hospital of Nanchang University, Nanchang, China

**Keywords:** cervical cancer, differentially expressed genes, immune infiltration, tumor immunity, tumor-infiltrating CD8^+^ T cells

## Abstract

Tumor-infiltrating immune cells, associated with tumor progression, are promising prognostic biomarkers. However, the relationship between levels of gene expression and that of immune cell infiltration in cervical cancer prognosis is unknown. In this study, three cervical cancer gene expression microarrays (GSE6791, GSE63678 and GSE55940) were obtained from the GEO database. The IDO1 gene was identified by differentially expressed gene screening. The gene expression profiles of TCGA and GTEx databases along with comprehensive bioinformatics analysis identified that the IDO1 gene was upregulated in cervical cancer with significant difference in expression at different N stages. In addition, it was also upregulated in HPV16 positive sample. The pan-cancer analysis identified that IDO1 was highly expressed in most cancers. TIMER analysis revealed that the expression of IDO1 in CESC shows positive correlation with CD8^+^ T cells, CD4^+^ T cells, neutrophils, dendritic cells. IDO1 expression showed remarkable positive correlation with all immune cell markers except M1 macrophages. CD8^+^ T cell infiltration GSEA results showed that IDO1 was mainly associated with tumor immune-related signaling pathways.

## Introduction

Cervical cancer is one of the most prevalent malignant diseases, affecting women worldwide (1). Persistent infection with high-risk human papillomaviruses (mainly HPV16 and HPV18) is the main cause of cervical cancer and its precursor lesions (2, 3). The incidence of cervical cancer has decreased due to the production of HPV vaccines, improved living conditions, and early screening (4). However, there is still a high mortality of cancer in Asia (5). Therefore, necessary measures are needed to reduce the incidence of cervical cancer. It is well known that the HPV-E6 and E7 genes encode two mucoproteins involved in the pathogenesis of cervical cancer. Cervical cancer is therefore a relatively immunogenic cancer that can use various mechanisms to evade immune attack by the host (6).

The interaction of tumor cells with the microenvironment plays a crucial role in the development of malignant tumors (7). The main component of the tumor microenvironment is the tumor-infiltrating lymphocytes, a specific population of T cells with a high specific immune response to tumor cells (8, 9). The immune microenvironment of cervical cancer not only has common features of solid tumors but also unique characteristics associated with HPV infection (10). The importance of infiltrating lymphocytes in predicting progression of different types of solid tumors has been demonstrated and T cells are the immune cells of choice for treating cancer. Tumor cell growth is attributed to develop immune resistance by evading immune surveillance mechanisms, leading to T cell dysfunction and depletion (11). Overcoming T-cell dysfunction in cancer patients is the focus of oncology treatment. Blocking antibodies against cytotoxic T-lymphocyte-associated protein 4 (CTLA-4) and PD-1/PD-L1 immune checkpoints have shown durable clinical responses in a variety of cancers, including cervical cancer (12–14). However, many cancer patients show primary or secondary treatment tolerance to immune checkpoint therapy (15). Therefore, the search for more immune checkpoint-related genes to reverse T-cell dysfunction is essential. Indoleamine 2, 3-dioxygenase 1 (IDO1) is characterized by a rate-limiting metabolic enzyme that converts tryptophan (Trp) to downstream Kyn (Kyn) (16, 17). IDO1 is interferon-induced and has been shown to mediate powerful immunosuppression in cancer. Growing evidence indicates that IDO1 is overexpressed in the vast majority of solid tumors and associated with clinical prognosis, such as anal (18), esophageal (19), as well as cervical cancer (20). More importantly, several molecular drugs targeting IDO1 have been evaluated in multiple clinical trials with encouraging results (21, 22).

In this study, three cervical cancer gene expression microarrays were screened for differentially expressed genes and their expression was analyzed in Cervical squamous cell carcinoma and endocervical adenocarcinoma (CESC). The significance of the genes in the prognosis of CESC was determined by correlating the level of gene expression with that of immune cell infiltration and immune checkpoint-related gene expression. The Gene Set Enrichment Analysis (GSEA) identified the immune-related signaling pathways in the tumor.

## Methods

### Data Sources

Gene expression data microarrays for cervical cancer were obtained from the Gene Expression Omnibus (GEO) database: GSE6791 (23) (containing 84 samples), GSE55940 (24) (containing 10 samples) and GSE63678 (25) (containing 35 samples).

The human cancer gene expression data and related clinical information were obtained from the The Cancer Genome Atlas (TCGA) and Genotype-Tissue Expression (GTEx) database. There were 10201 tumor samples in 33 tumors from TCGA database and 16871 normal samples from TCGA+GTEx database.

### Differentially Expressed Gene

The”affy” and the “impute” R packages in R/Bioconductor software were used for GEO data processing, and then limma package of R software (version 3.40.2) was used for the analysis of differential gene expression. To correct false-positive results, adjusted P values were analyzed in TCGA or GTEx. Differential gene screening was performed based on adjusted P < 0.05, |logFC| > 1 and volcano plotting was performed using the ggplot2 R package. Overlapping genes were searched by VennDiagram package.

### Immunocorrelation Analysis

The TIMER database (http://timer.comp-genomics.org/) was used to analyze the relation of IDO1 expression in CESC with the level of immune cell infiltration and immune cell markers. P<0.05 was considered statistically significant. Immune scores were assessed using the CIBERSORT algorithm. CIBERSORT is a versatile computational method for quantifying cell fractions from bulk tissue gene expression profiles (26). Spearman correlation test was used for the analysis of the correlation between gene and immune checkpoint associated gene. Visualization was achieved by the R (v4.0.3) package ggplot2.

### Survival Analysis

Gene expression and immune cell infiltration levels were analyzed by the TIMER database in relation to overall survival (OS). Kaplan-Meier test was performed to analyze the difference between the survival of patients with high and low gene expression or high and low infiltration levels.

### Protein Expression Validation

Immunohistochemical staining maps of CD8+ T cells markers for protein expression in both cervical cancer tissue and normal tissue were downloaded from The Human Protein Atlas (HPA) database.

### Gene Set Enrichment Analysis

The samples were divided into two groups of high and low expression according to the median value of gene expression level, the group cutoff was set to median (Cutoff‐High and Cutoff‐Low are both 50%). GSEA was performed to investigate the functions correlated with different risk groups by GSEA 4.1.0, and the software was downloaded from the website for GSEA (http://www.gseamsigdb.org/gsea/downloads). The screening conditions were |NES|≥ 1, FDR < 0.25 and P < 0.05.

## Results

### Screening of Differentially Expressed Genes

**Figure 1:**
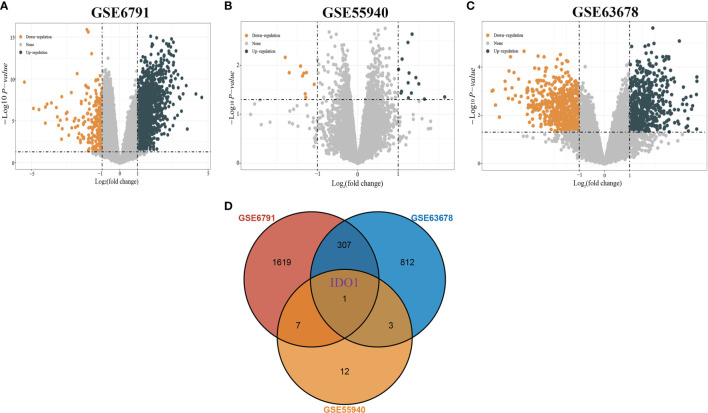
Screening of differentially expressed genes. [**(A)** Volcano plot of DEGs in GSE6791; **(B)** Volcano plot of DEGs in GSE55940; **(C)** Volcano plot of DEGs in GSE63678; **(D)** Venn diagram showing overlapping genes of DEGs in the three datasets].

Analytical screening of the GSE6791, GSE55940, and GSE63678 datasets yielded 1698, 14, and 540 differentially up-regulated genes and 236, 9, and 583 differentially down-regulated genes respectively (**Figures 1A–C**). Overlapping gene analysis of the differentially expressed gene identified a key gene IDO1 (**Figure 1D**).

### Expression of IDO1 in CESC and Multiple Cancer

**Figure 2:**
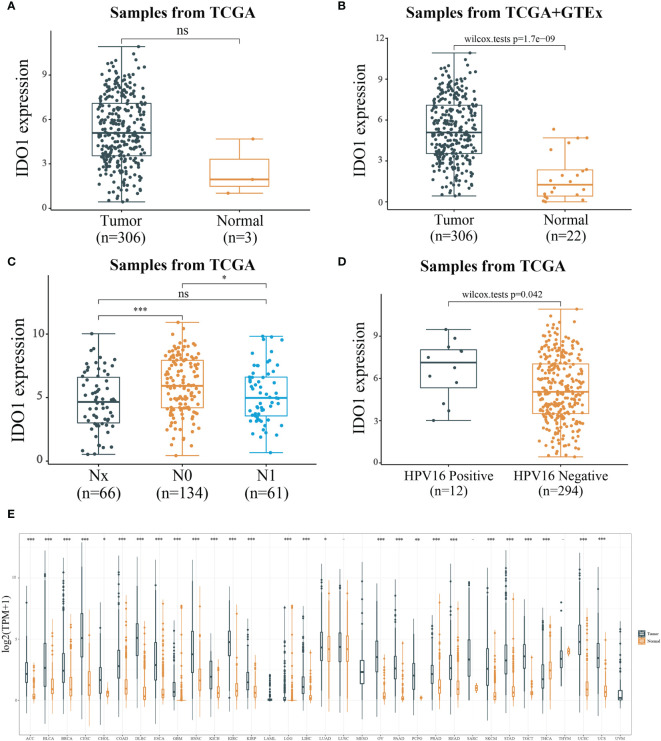
Expression level of IDO1. [**(A)** IDO1 expression level in cancerous and paraneoplastic tissues of CESC from TCGA datasets; **(B)** IDO1 expression level in cancerous and normal tissues of CESC from TCGA+GTEx datasets; **(C)** IDO1 expression level in different N stages of CESC; **(D)** IDO1 expression level in CESC with or without HPV type 16 infection; ns: P > 0.05, *P < 0.05, ***P < 0.001].

IDO1 expression in CESC and paraneoplastic tissues was analyzed through TCGA database (306 tumor samples and 3 adjacent tumor samples) using Wilcox-tests in R software. No significant difference was observed in IDO1 expression (**Figure 2A**), probably because of the small number of adjacent tissue samples. IDO1 expression data was compared by integrating GTEx normal tissues which counts for 22 normal tissue. It was found that IDO1 expression was significantly upregulated in CESC compared to normal tissues (**Figure 2B**). The samples with unknown staging information were eliminated, there were 66 Nx samples, 134 N0 samples and 61 N1 samples from TCGA database. Analysis of IDO1 expression at different N stages revealed that IDO1 expression was highest in the N_0_ stage (**Figure 2C**). There were12 HPV-16 positive samples and 294 HPV-16 negative samples from TCGA database. IDO1 expression was remarkably higher in HPV16 positive than HPV16 negative samples (**Figure 2D**). Subsequently, tumor samples in TCGA were integrated with normal samples in GTEx (10201 tumor samples and 16871 normal samples from TCGA+GTEx database). The pan-cancer expression profile of IDO1 was analyzed using rank sum test. It was observed that IOD1 was remarkably down-regulated in Thyroid carcinoma (THCA) and up-regulated in the remaining cancers except for Acute Myeloid Leukemia (LAML), Lung squamous cell carcinoma (LUSC), Mesothelioma (MESO), Sarcoma (SARC), Thymoma (THYM), and Uveal Melanoma (UVM) (**Figure 2E**).

### Immune Cell Infiltration Correlation

**Figure 3:**
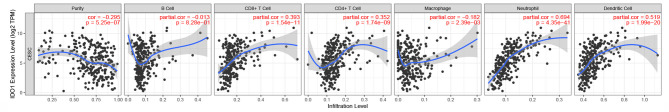
Correlation between IDO1 expression level and the level of immune cell infiltration.

IDO1 expression levels in CESC were negatively correlated with tumor purity and macrophages but positively correlated with CD8^+^ and CD4^+^ T cells, neutrophils, and dendritic cells (**Figure 3**). The relationship between IDO1 expression and different immune cell type markers was further analyzed. By purity-adjusted correlation, it was identified that IDO1 expression levels show significant positive correlation with all immune cell markers except M1 macrophages (**Table 1**). The results showed that although IDO1 expression level had no correlation with B cell infiltration it had significant correlation with the type of B cell markers. This suggests that the correlation between IDO1 expression level and B cell infiltration could be verified by the expression of IDO1 and its type markers.

**Table 1:** Correlation analysis between IDO1 and markers of immune cells in TIMER.

Description		Gene markers	IDO1			
			None		Tumor purity	
			Cor	P	Cor	P
CD8+ T cell		CD8A	0.678	0.00E+00	0.651	7.80E-35
		CD8B	0.508	0.00E+00	0.466	2.41E-16
T cell(general)		CD3D	0.637	0.00E+00	0.599	2.18E-28
		CD3E	0.659	0.00E+00	0.623	3.83E-31
		CD2	0.648	0.00E+00	0.606	3.83E-29
B cell		CD19	0.343	7.19E-10	0.235	7.59E-05
		CD79A	0.332	3.31E-09	0.202	7.16E-05
Monocyte		CD86	0.569	0.00E+00	0.514	4.10E-20
		CD115	0.477	0.00E+00	0.392	1.26E-11
TAM		CCL2	0.266	2.47E-06	0.169	4.91E-03
		CD68	0.303	7.65E-08	0.240	5.62E-05
		IL10	0.346	5.17E-10	0.267	6.78E-06
M1 Macrophage		INOS	0.082	1.53E-01	0.066	2.76E-01
		IRF5	0.116	4.24E-02	0.105	8.08E-02
		COX2	0.011	8.51E-01	-0.038	5.25E-01
M2 Macrophage		CD163	0.425	0.00E+00	0.356	1.08E-09
		VSIG4	0.349	4.61E-10	0.287	1.19E-06
		MS4A4A	0.460	0.00E+00	0.394	1.04E-11
Neutrophils		CD66b	0.002	9.73E-01	0.012	8.37E-01
		CD11b	0.376	1.41E-11	0.321	4.85E-08
		CCR7	0.386	2.47E-12	0.316	7.92E-08
Natural killer cell		KIR2DL1	0.348	3.93E-10	0.301	3.28E-07
		KIR2DL3	0.469	3.63E-18	0.426	1.27E-13
		KIR2DL4	0.548	1.97E-25	0.523	7.02E-21
		KIR3DL1	0.408	1.02E-13	0.331	1.58E-08
		KIR3DL2	0.461	1.57E-17	0.407	1.68E-12
		KIR3DL3	0.339	1.18E-09	0.275	3.41E-06
		KIR2DS4	0.376	1.00E-11	0.355	1.23E-09
Dendritic cell		HLA-DPB1	0.602	0.00E+00	0.574	1.08E-25
		HLA-DQB1	0.501	0.00E+00	0.476	4.65E-17
		HLA-DPA1	0.643	0.00E+00	0.623	3.94E-31
		BDCA-1	0.259	4.52E-06	0.196	1.02E-03
		BDCA-4	0.048	4.07E-01	-0.024	6.89E-01
		CD11c	0.460	0.00E+00	0.376	1.01E-10
Th1		T-bet	0.658	2.12E-39	0.616	2.27E-30
		STAT4	0.490	7.08E-20	0.413	7.54E-13
		STAT1	0.681	0.00E+00	0.656	1.68E-35
		IFN-γ	0.654	9.57E-39	0.620	8.43E-31
		TNF-α	0.182	1.42E-03	0.137	2.23E-02
Th2		GATA3	0.247	1.27E-05	0.217	2.72E-04
		STAT6	0.174	2.21E-03	0.160	7.56E-03
		STAT5A	0.394	8.50E-13	0.402	3.34E-12
		IL13	0.211	2.06E-04	0.126	3.66E-02
Th17		BCL6	0.026	6.56E-01	0.042	4.87E-01
		IL21	0.360	8.76E-11	0.302	2.90E-07
		STAT3	0.182	1.41E-03	0.165	5.84E-03
		IL17A	0.133	1.95E-02	0.126	3.64E-02
Treg		FOXP3	0.521	0.00E+00	0.453	2.00E-15
		CCR8	0.428	4.63E-15	0.360	7.06E-10
		STAT5B	0.022	6.99E-01	0.034	5.73E-01
		TGFβ	0.120	3.66E-02	0.031	6.13E-01
T cell exhaustion		PD-1	0.628	0.00E+00	0.590	2.19E-27
		CTLA4	0.615	0.00E+00	0.564	1.07E-24
		LAG3	0.636	0.00E+00	0.594	8.06E-28
		TIM-3	0.612	0.00E+00	0.574	1.22E-25
		GZMB	0.613	0.00E+00	0.574	1.25E-25

### Immunoscore

**Figure 4:**
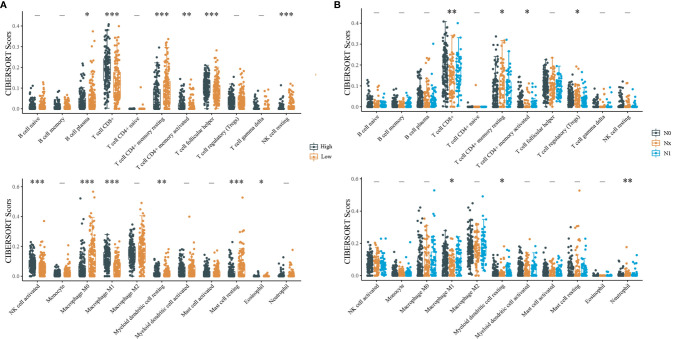
CIBERSORT statistics of relative proportions of immune cell subpopulations. [**(A)** Distribution of immune scores for different IDO1 expression level; **(B)** Distribution of immune scores for different N stages; “-”P > 0.05, *P < 0.05, **P < 0.01, ***P < 0.001].

The relative proportion of immune cell subsets in CESC samples with different IDO1 expression and different N stages in the TCGA dataset were counted using CIBERSORT. According to the median value of IDO1 expression, samples were divided into high and low expression groups. In **Figure 4A**, it can be seen that CD8^+^ T cells and M0 macrophages account for the majority of the 22 tumor-infiltrating immune cell subsets. **Figure 4B** shows that CD8^+^ T cells account for the highest proportion, revealing that the level of CD8^+^ T cell infiltration was significantly correlated with IDO1 expression and different N stages of CESC.

### Relationship Between CD8+ T-Cell Infiltration and Prognosis of CESC

**Figure 5:**
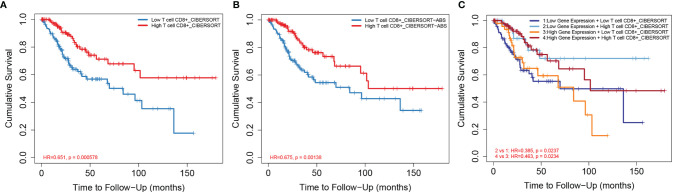
Relationship between CD8+ T-cell infiltration and prognosis of CESC. [**(A)** CD8+ T-cell infiltration level and CESC prognosis based on CIBERSORT algorithm; **(B)** CD8+ T-cell infiltration level and CESC prognosis based on CIBERSORT-ABS algorithm; **(C)** CD8+ T-cell infiltration level and IDO1 expression level and CESC prognosis based on CIBERSORT algorithm].

Based on the median ratio of CD8^+^ T-cell infiltration and IDO1 expression, we divided the patient population into two groups (low and high ratios). By analyzing different CD8^+^ T-cell infiltration levels and CESC prognosis, it was found that patients with high CD8^+^ T-cell infiltration levels had a better prognosis as verified by CIBERSORT (**Figures 5A, B**), XCELL and QUNATISEQ (**Supplementary Figure 1**); low CD8^+^ effector memory T-cell infiltration levels were significantly associated with poor prognosis of patients. Further, the effects of both, the IDO1 expression and CD8^+^ T cell infiltration together were compared to patient prognosis (**Figure 5C**). It revealed that patients with high CD8^+^ T cell infiltration levels had a better prognosis in the case of high IDO1 expression. The prognosis of patients with high CD8^+^ T-cell infiltration levels was better with low IDO1 expression. CD8^+^ T-cell infiltration level can not only be regulated by IDO1 expression levels but also by other biological mechanisms in tumor microenvironment. Thus, co-influence of IDO1 expression level and CD8+ T-cell infiltration level on the prognosis of patients might be different from their individual impact.

### Expression of Immune Checkpoint-Related Genes

**Figure 6:**
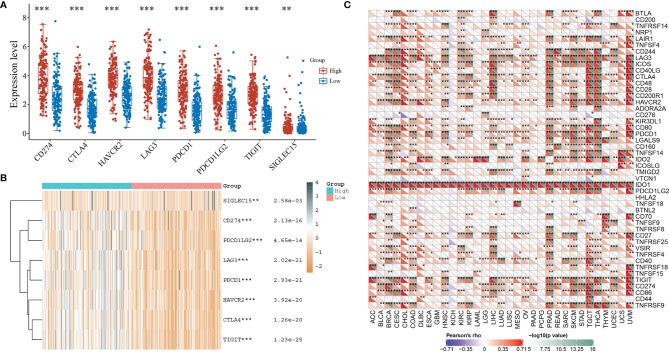
Correlation between IDO1 expression level and immune checkpoint-associated gene expression. [**(A)** Immune checkpoint-associated gene expression level in high and low expression level of IDO1 samples; **(B)** Heat map of immune checkpoint-associated gene expression; **(C)** Immune checkpoint-associated gene expression level in multiple cancers; *P < 0.05, **P < 0.01, ***P < 0.001].

IDO1 expression was classified as high or low according to the median expression value. The expression of immune checkpoint-related genes in high and low expression IDO1 were observed. The results indicate that the IDO1 expression shows significant positive correlation with the expression of immune checkpoint-related genes (**Figures 6A, B**). The correlation of expression level between IDO1 and other immune checkpoint-related genes in cancers are displayed in **Figure 6C**. Analysis of immune checkpoint-related gene expression, in multiple cancers, revealed that IDO1 itself is an immune checkpoint gene.

### Validation of the Protein Expression of CD8+ T Cells Markers

**Figure 7:**
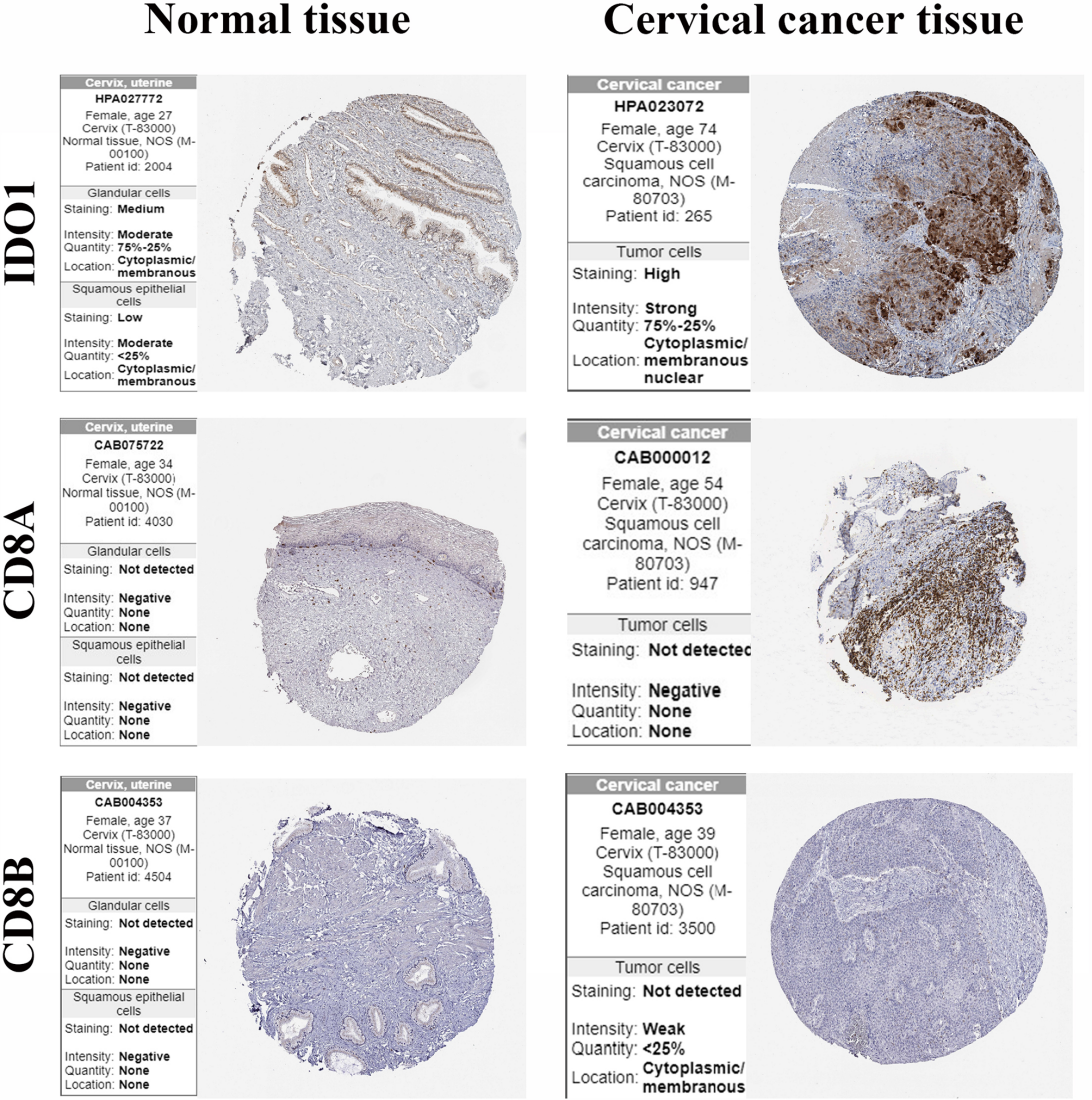
Protein expression of IDO1 and gene markers in cervical cancer tissue and normal tissue.

The protein expression of IDO1 and CD8+ T cells markers in cervical cancer tissues and normal cervical tissues was verified using the HPA online database (**Figure 7**). The results showed that CD8A and CD8B were not detected in both cancer tissues and normal liver tissues. IDO1 was highly detected in cervical cancer tissue and low expression in normal tissue.

### Gene Set Enrichment Analysis

IDO1 expression was classified as high or low according to the median expression value. Red bar in Figures indicated low expression level while blue bar was high expression level.

**Figure 8:**
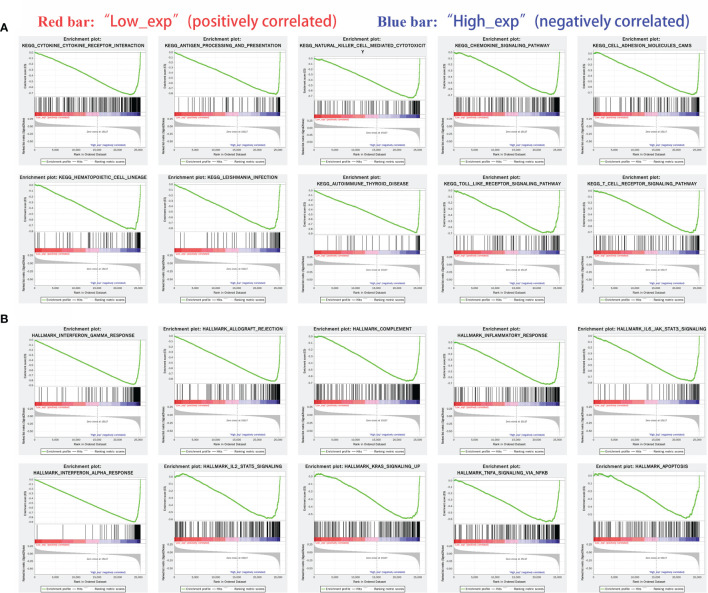
Gene enrichment analysis of IDO1 in cancers. [**(A)** Highly expressed IDO1 in KEGG enriched top ten pathway; **(B)** Highly expressed IDO1 in HALLMARK enriched top ten pathway].

KEGG pathway analysis showed that: cytokine and cytokine receptor interaction channels, antigen presentation and natural killer cell-mediated cytotoxicity channels were three significantly different pathways (**Figure 8A**). HALLMARK pathway analysis showed that the interferon-gamma response, allograft rejection and complementation were the three most significant pathways (**Figure 8B**).

(KEGG: chemokine signaling pathway, cell adhesion molecules, hematopoietic cell lineage, Leishmania protozoa infection, autoimmune thyroid disease and TOLL-like receptor signaling pathway

HALLMARK: inflammatory response, IL6/JAK/STAT3 signaling pathway, interferon-alpha response, IL2-STAT5, KRAS signaling, TNF-alpha/NF-kb and apoptosis)

## Discussion

Tumor cells are malignantly transformed normal cells. There are many mutated or aberrantly expressed proteins that may serve as tumor antigens (27). By recognizing these tumor antigens, T cells can clear tumor cells. Thus, the T cell immune response against tumor antigens is a central mechanism of the body’s anti-tumor immunity. Studies indicate that the level of tumor-infiltrating lymphocytes is positively correlated with the clinical prognosis of patients in a variety of solid tumors, thus demonstrating the existence of tumor immune surveillance (28–30).

In this study, we identified the IDO1 gene, by screening three cervical cancer gene expression microarrays for DEGs. These DEGs were upregulated in cervical cancer with significant difference in expression at different N stages and HPV-16 infection. Studies have shown that IDO1 is overexpressed in many solid tumor tissues. A study reported that IDO1 is with higher mRNA transcription and protein expression level than in normal cervix, and also in comparison to other cancers. We confirmed this conclusion by pan-cancer analysis (31). IDO1 is a rate-limiting enzyme that converts tryptophan to kynurenine. It plays a role in immunosuppression by increasing tryptophan metabolism in the tumor microenvironment (32). IDO1 expression may be induced by the secretion of IFN-γ by CD8^+^ T cells in the tumor microenvironment. Elevated IDO1 expression produces a series of effects such, as inhibition of: T cell function, CD4^+^ T cell differentiation pathway to T_reg_, and antigen presentation (33, 34). Tumors can utilize multiple escape mechanisms to avoid immune recognition. For antigen presentation, firstly, the antigen must be taken up by the dendritic cells and presented to CD8^+^ T cells. Secondly, the antigen must have direct tumor presentation in order to be recognized and killed by activated CD8^+^ T cells (35). These two processes are where immune escape occurs. The immune escape mechanisms include, regulation of antigen expression and alteration of antigen processing and presentation mechanisms in tumor cells (36, 37).

Tumor-infiltrating immune cells are major contributors to the tumor immune response. Their levels predict treatment outcome and survival (38). We found that in CESC, IDO1 expression levels were significantly correlated with the levels of infiltrating B cells, macrophages, CD8^+^ T cells, CD4^+^ T cells, neutrophils, and dendritic cells. Further analysis revealed that IDO1 expression level shows remarkable positive correlation with all immune cell markers except M1 macrophages. CD8^+^ T cells are a major component of tumor-infiltrating lymphocytes. Therefore, regulation of CD8^+^ T cell responses has been a focus of immunotherapy for cancer (39). It has been shown that a large infiltration of CD8^+^ T cells is associated with good prognosis in some tumors (40–42). In the present study, we found the highest and most significant proportion of CD8^+^ T cells in samples with different IDO1 expression and at different N stages by the CIBERSORT algorithm. We further observed the correlation between the CD8^+^ T cell infiltration and patient prognosis in CESC by different algorithms. The results showed that high CD8^+^ T-cell infiltration was remarkably associated with good prognosis in CESC patients. Combined with the expression level of IDO1, the prognosis of patients with low IDO1 expression was better at a high CD8^+^ T cell infiltration level. From this perspective, we analyzed the relationship between IDO1 expression and immune checkpoint-related genes. The results indicated that the IDO1 expression was significantly correlated with immune checkpoint-related gene expression. This suggests that IDO1 is itself an immune checkpoint gene. The expression of IDO1 creates two effects on its own microenvironments. It decreases tryptophan(Trp) and produces a series of toxic kynurenine(Kyn) metabolites (43). The toxic Kyn metabolites directly suppress the effector T cell response by favoring differentiation of Tregs (44, 45). The immune checkpoint is a key molecule in T-cell dysfunction. Hence, we can reverse T-cell dysfunction by regulating the expression of immune checkpoint molecules. This means that IDO1, which is an immune checkpoint gene is an important target for cancer immunotherapy intervention (46) (47). first reported the results of the phase I clinical trial of Epacadostat, a selective IDO1 inhibitor, for the treatment of advanced malignant solid tumors. The results show that Epacadostat was well tolerated and effectively inhibited IDO1 activity and Kyn levels. In recent years, IDO1 inhibitors have been used as a promising immunomodulatory agent for patients with advanced cancer (48–50). In practice, the infiltrated CD8^+^/T_reg_ ratio may be a more precise parameter for the prognosis. Jallad et al. found that the triple immune therapy was capable of significantly enhancing the natural killer cell counts as well as the CD3^+^CD4^+^/T_reg_ and CD3^+^CD8^+^/Treg ratios possibly enhancing the anti-tumorigenic environment (44). The present study also provides support for the application of IDO1 inhibitors in cervical cancer. Finally, to verify the possible mechanism of action of IDO1, the results of KEGG and HALLMARK enrichment analysis showed that IDO1 was mainly enriched in cytokine and cytokine receptor interaction, antigen presentation and natural killer cell-mediated cytotoxicity, and interferon γ response channels. This suggests that IDO1 is a tumor immunity and tumor escape related gene in CESC and can be used as a new target for cervical cancer therapy.

## Conclusion

In summary, Indolamine-2,3-dioxygenase-1 (IDO1) is a cytosolic enzyme that catalyzes the conversion of+ essential amino acid Trp to kynurenine (Kyn). IDO1 is overexpressed in more than 50% of tumors and its overexpression increases the relative concentration of Kyn compared to Trp. Hence Kyn/Trp ratio can be used as a prognostic marker to monitor cancer invasiveness and progression. Our study mainly found that IDO1 can be a biomarker for prognosis prediction in CESC and was closely associated with infiltrating CD8+ T cells and immune checkpoint genes. This study provides ideas for the application of IDO1 inhibitors in the treatment of CESC and explores the potential value to enhance anti-tumor immunity and immunotherapy.

## Data Availability Statement

The datasets presented in this study can be found in online repositories. The names of the repository/repositories and accession number(s) can be found in the article/**Supplementary Material**.

## Author Contributions

All authors (SZ, JW, MC, DC, JX, QC) statement: (1) SZ and JW as co-first author contributed ueqially to this work. (2) QC as corresponding author designed the whole project and provided financial support.

## Funding

Funding was received from the following: General program of NSFC, 81560247, PPAR γ Molecular mechanism of regulating mat2a gene transcription in the pathogenesis of endometriosis; Scientific and technological research project of Jiangxi Provincial Department of education, GJJ180006, molecular regulation mechanism of ATAD3A/WASF3/KiSS1 signaling pathway in the pathogenesis of cervical cancer; Science and technology plan of Jiangxi Provincial Health and Family Planning Commission, 20185124, molecular mechanism of JAK2/STAT3 signaling pathway regulating WASF3 gene transcription on cervical cancer.

## Conflict of Interest

The authors declare that the research was conducted in the absence of any commercial or financial relationships that could be construed as a potential conflict of interest.

## Publisher’s Note

All claims expressed in this article are solely those of the authors and do not necessarily represent those of their affiliated organizations, or those of the publisher, the editors and the reviewers. Any product that may be evaluated in this article, or claim that may be made by its manufacturer, is not guaranteed or endorsed by the publisher.

## Supplementary Material

Supplementary Figure 1 Relationship between CD8+ T-cell infiltration and prognosis of CESC *via* different algorithm. **(A)** CD8+ T-cell infiltration level and CESC prognosis based on xCELL algorithm; **(B)** CD8+ infiltration level and CESC prognosis based on QUANTISEQ algorithm; **(C)** CD8+ effector memory infiltration level and CESC prognosis based on xCELL algorithm. T-cell infiltration level and CESC prognosis.

Supplementary Table 1 Genes in Venn diagram
