# Supplementary figures and images for: Tumor-Infiltrating CD8+ T Cells Driven by the Immune Checkpoint-Associated Gene IDO1 Are Associated With Cervical Cancer Prognosis
Source: Front Oncol. 2021 Oct 27;11:720447. doi: 10.3389/fonc.2021.720447 (PMC8578845; doi:10.3389/fonc.2021.720447)

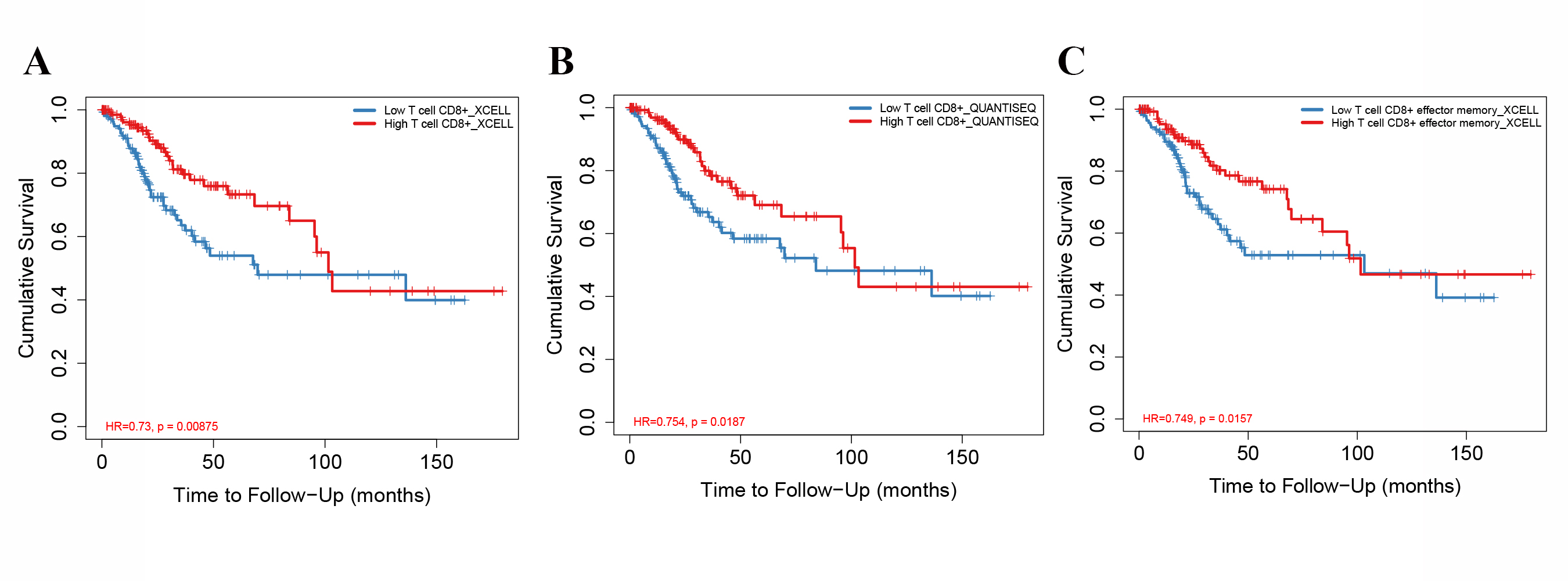

Supplement: Supplementary Figure 1 — Relationship between CD8+ T-cell infiltration and prognosis of CESC via different algorithm. (A) CD8+ T-cell infiltration level and CESC prognosis based on xCELL algorithm; (B) CD8+ infiltration level and CESC prognosis based on QUANTISEQ algorithm; (C) CD8+ effector memory infiltration level and CESC prognosis based on xCELL algorithm. T-cell infiltration level and CESC prognosis. [file Image_1.jpeg]
